# Budgeting and Advocacy to Improve Water, Sanitation, and Hygiene in Health Care Facilities: A Case Study in Nepal

**DOI:** 10.9745/GHSP-D-23-00491

**Published:** 2024-06-27

**Authors:** Laxman Kharal Chettry, Prakash Bohara, Ramesh C. Bohara, Ramhari Rijal, Sarad Khadha, Hari Subedi, Debesh Giri, Sarbesh Sharma, Upendra Dhungana, Matteus van der Valen, John Brogan, Darcy M. Anderson

**Affiliations:** a Terre des hommes Foundation, Nepal Country Office, Lalitpur, Nepal.; b Swiss Water and Sanitation Consortium, Zurich, Switzerland.; c Thakurbaba Municipality, Nepal.; d Geruwa Rural Awareness Association, Gulariya Municipality, Nepal.; e Management Division, Department of Health Services, Ministry of Health and Population, Kathmandu, Nepal.; f Helvetas, Zurich, Switzerland.; g The Water Institute, Gillings School of Global Public Health, University of North Carolina at Chapel Hill, Chapel Hill, NC, USA.

## Abstract

Barriers to achieving and sustaining access to water, sanitation, hygiene, cleaning, and waste management (WASH) in health care facilities include a lack of supportive policy environment and adequate funding. While guidelines exist for assessing needs and making initial infrastructure improvements, there is little guidance on how to develop budgets and policies to sustain WASH services. We conducted costing and advocacy activities in Thakurbaba municipality, Nepal, to develop a budget and operations and maintenance policy for WASH in health care facilities in partnership with the municipal government. Our objectives for this study were to (1) describe the process and methods used for costing and advocacy, (2) report the costs to achieve and maintain basic WASH services in the 8 health care facilities of Thakurbaba municipality, and (3) report the outcomes of advocacy activities and policy development. We applied bottom-up costing to enumerate the resources necessary to achieve and maintain basic WASH services and their costs. The annual costs to achieve, operate, and maintain basic access to WASH services ranged from US$4881–US$9695 per facility. Cost findings were used to prepare annual budgets recommended to achieve, operate, and maintain basic services, which were presented to the municipal government and incorporated into an operations and maintenance policy. To date, the municipality has adopted the policy and established a recovery fund of US$3831 for repair and maintenance of infrastructure and an additional US$153 per facility for discretionary WASH spending, which were to be replenished as they were spent. Advocacy at the national level for WASH in health care facilities is currently being championed by the municipality, and findings from this project have informed the development of a nationally costed plan for universal access. This study is intended to provide a roadmap for how cost data can be collected and applied to inform policy.

## Abstract (precis)

We demonstrated that costing and advocacy can be successfully used to establish budgets and policies for sustainable operations and maintenance of water, sanitation, and hygiene in health care facilities in Thakurbaba municipality, Nepal, as part of progressing toward universal access.

## Teaser

**Key Messages:**

- Developing and allocating funding for operations and maintenance for basic water, sanitation, hygiene, cleaning, and waste management (WASH) services in health care facilities remains a challenge in Nepal.
- We estimated annualized costs across 8 health care facilities in Thakurbaba municipality and found that additional investment (US$1659–US$4285) per facility per year was needed to achieve basic service.
- We used dissemination workshops, stakeholder consultations, and data validation and certification processes recommended by the municipality to build credibility and trust in the budget estimates.
- Based on budget estimates, Thakurbaba municipality successfully drafted, adopted, and funded an evidence-based policy for WASH in health care facilities operations and maintenance and is advocating at the provincial and national levels for funding and costed roadmaps.

**Key Implication:**

- We describe the process of costing, policy development, and advocacy to serve as a roadmap for policymakers and practitioners to progress toward universal access, in line with the Eight Practical Steps recommended by the World Health Organization and UNICEF for WASH in health care facilities.

## INTRODUCTION

Environmental conditions for water, sanitation, hygiene, cleaning, and waste management (WASH) in health care facilities are critical for safe care delivery and a well-functioning health system. In recognition of the importance of these conditions, the World Health Organization (WHO) and UNICEF have published guidelines for 8 recommended steps for countries to achieve universal coverage.^1^ These guidelines—often called the “Eight Practical Steps”—have been widely adopted as a framework for guiding national action, and the WHO and UNICEF track progress on these steps for over 70 countries.^2^ The first steps are conducting situation assessments, setting targets, and developing national costed roadmaps for achieving those targets, which are considered key preparations before widespread program implementation.

Nepal has begun implementing the Eight Practical Steps, starting with situation assessments and setting targets. In 2021, data from the WHO and UNICEF showed that 94% of health care facilities had an improved water source, 89% had improved sanitation, 97% had hand hygiene facilities at points of care, and 1% followed waste management procedures for safe segregation, treatment, and disposal.^3^ In 2018, the government of Nepal released draft national standards for WASH in health care facilities,^4^ which were broadly aligned with indicators recommended and used to measure basic and advanced access under the Joint Monitoring Program (JMP) of the WHO and UNICEF.^5^^,^^6^ The government formally endorsed these standards in 2021. Subsequent steps for developing a costed roadmap for achieving these standards are in progress as of 2024; a draft exists but is yet to be approved.

Funding for WASH in health care facilities in Nepal is disbursed from the federal government to the provincial level, which is subsequently disbursed to municipalities. Some funding is earmarked for specific major infrastructure projects (e.g., construction of new health care facilities). However, a substantial portion of funding is for discretionary spending, and municipal governments have broad authority to allocate this funding based on local needs and priorities.^7^ Under Nepal’s federal government system and relatively recent constitution—adopted in 2015—municipal governments also hold considerable authority for policymaking. The Local Governance Operation Act of 2017 gives municipalities the authority to adopt acts, regulations, and working procedures per their specific needs, including WASH service delivery.^8^ However, technical expertise among municipal governments for planning and budgeting is often low, and nongovernmental organizations (NGOs) play an important role in providing technical support and evidence to inform decision-making.^9^^–^^11^

In 2022, a Swiss-based NGO, Terre des hommes Nepal, conducted costing and advocacy activities in Thakurbaba municipality with the aim of accompanying the municipality to create budgets and an operations and maintenance (O&M) policy for WASH in health care facilities. As of 2024, these activities have successfully generated budgets for annual operating costs (and, where necessary, additional investment needed to reach basic service), which have been integrated into a policy for O&M. The municipal government has formally adopted the policy, allocated funding for its implementation, and begun deploying those funds to improve O&M. These outcomes are a meaningful achievement for municipal governments under the relatively new federalist government system and an important step for capacity-building and systems strengthening. Cost estimates and learnings from these activities have informed national-level advocacy and contributed to ongoing efforts to develop nationally costed roadmaps for WASH in health care facilities. In this study, we report on these activities. This article is intended to provide a case study for how cost data can inform advocacy and policymaking for WASH in health care facilities, particularly for executing the Eight Practical Steps.

Costing and advocacy activities have successfully generated budgets for annual operating costs, which have been integrated into an O&M policy for WASH in health care facilities in Thakurbaba municipality, Nepal.

Our study objectives were to (1) describe the process and methods used for budgeting and advocacy, (2) assess the costs of achieving and sustaining basic WASH services in the 8 health care facilities of Thakurbaba municipality, and (3) report the outcomes of policy development and advocacy activities. In the methods, we describe the key activities for data collection and advocacy. In the results, we report the data from costing and the outcomes from policy development and advocacy activities. In the discussion, we reflect on lessons learned and how this process may be applied in other contexts.

## METHODS

### Overview of Data Collection and Advocacy Activities

Costing, policy development, and advocacy activities were carried out in stages: (1) municipality-level advocacy, (2) tool development, (3) initiation workshop, (4) preliminary costing, (5) sharing and discussion workshop, (6) data validation and certification, (7) budget calculations, (8) O&M policy finalization and approval, and (9) dissemination and advocacy for scale-up.

These activities were focused on achieving and maintaining basic WASH services. We defined basic service as meeting the basic indicators outlined by the WHO and UNICEF under the JMP.^1^ However, health care facilities also included a small number of additional items not included under JMP indicators for basic service (e.g., fencing) or that went beyond basic to advanced service levels (e.g., water quality testing and treatment, additional toilets and hand hygiene facilities) that they considered necessary to provide adequate WASH. The Supplements indicate all included line items.

Our final list of WASH services included in costing and policy development were: water (source and pump, pipe network, water tower or storage system, and treatment); sanitation (toilets and associated hand hygiene facilities, and septic or other containment systems); hygiene (hand hygiene facilities at points of care); environmental cleaning; waste management (sluice room or other waste storage and processing area, autoclave, waste pit, placenta pit, and drainage); and fencing.

### Setting

This study was conducted in Thakurbaba municipality, in the Lumbini province of western Nepal. Thakurbaba municipality contains 10 health care facilities, 1 in each of its 9 wards and the Neulapur Municipal Hospital. We excluded 2 from this study: the Neulapur Hospital, which was under construction at the time of this research, and the Godana Basic Health Center, which is located in the government forestry office and does not have its own WASH infrastructure. Demographic information on the 8 included health care facilities is provided in Table 1.

**TABLE 1.** Characteristics of Health Care Facilities Included in the Study Sample, Thakurbaba Municipality, Nepal

**Facility Name**	**Services Offered**	**Deliveries/Month, No.**	**Outpatient Visits/Month, No.**
Neulapur Health Post	Outpatient, maternity, laboratory	25	1,418
Bagnaha Health Post	Outpatient, maternity, laboratory	8	666
Shivapur Health Post	Outpatient, maternity, laboratory	16	667
Thakurdwara Health Post	Outpatient, maternity, laboratory	24	297
Ranipur Basic Health Center	Outpatient	0	193
Bankatti Basic Health Center	Outpatient	0	328
Mohanpur Basic Health Center	Outpatient	0	425
Thakurdwara Basic Health Center	Outpatient	0	210

### Stage 1: Municipality-Level Advocacy

We organized a targeted workshop attended by the mayor, deputy mayor, chief administrative officer, and chief of health section of Thakurbaba municipality and the heads of local health care facilities. During the workshop, we presented the current status of WASH, with photographs showcasing the deteriorated conditions at various health care facilities within the municipality. Our messages emphasized the urgent need for policy development and enhanced O&M practices. Our presentations visually and verbally conveyed the critical gaps in current WASH facilities that had implications for public health and safety. As the outcome of this advocacy workshop, the municipality recognized the critical nature of the issues presented, expressed their interest in collaborating to establish a policy framework that could support sustained improvements, and formally requested our assistance in developing an initiative to improve the O&M of WASH at their health care facilities.

### Stage 2: Tool Development

We created a costing tool in Microsoft Excel based on previously developed methods.^12^^–^^14^ We chose Excel in part because project personnel were familiar with it, and the tabular format was well suited to data entry. We used bottom-up costing, in which all resources used in WASH provision were enumerated, a quantity and unit price were estimated for each resource, and total costs were calculated based on quantities and unit price. We prepopulated the spreadsheet with resources essential for achieving basic WASH, based on previous research.^14^ We conducted a pilot visit to 1 health care facility, where we observed conditions and availability of infrastructure, goods, and services related to WASH. We also conducted a meeting to understand how repair tasks were carried out. This information was used to refine the list of resources prepopulated in the spreadsheet.

The final spreadsheet contained costs for initial capital investments (hardware and software) and annual O&M (maintenance, personnel, recurrent training, consumables). Table 2 provides definitions and examples for each cost type.

**TABLE 2.** Categories of WASH Expenses Included in Costing Study

**Cost Category**	**Definition**	**Example Included Expenses**
Capital hardware	Infrastructure or equipment purchases required to establish WASH services or implement changes to service delivery method that are not consumed during normal service operation.	Sanitation facilities (superstructure with squat pan/seats, pit/septic tank) Water source and pipe network
Capital software	Planning, procurement, and/or initial training costs associated with establishing new WASH services or implementing changes to WASH service delivery method.	Initial infection prevention and operations and maintenance trainings delivered upon establishing infrastructure
Maintenance	Expenses required to repair and maintain functionality of capital hardware, including labor costs required for these purposes.	Breakdown repairs (e.g., clogged pipes) Cleaning of toilets, patient care areas Supplies for water system testing (e.g., arsenic, residual chlorine)
Recurrent software	Necessary trainings, behavior change, and other nontangible produced to be delivered each year for the upkeeping of the established and other introduced practices.	Annual infection prevention training Annual WASH FIT meetings
Personnel	Labor costs associated with normal operation of a service, including staff benefits; labor costs for maintenance (e.g., plumbers and repair technicians that are outsourced) are included under maintenance.	WASH focal person Support staff
Consumables	Products and supplies that are consumed during normal operation.	Handwashing soap Cleaning detergents Cleaning tools (e.g., mops, brooms)
Support	Expenses required to strengthen WASH provision but that do not have direct service outputs.	Communication Capacity-building

Abbreviations: WASH, water, sanitation, hygiene, cleaning, and waste management; WASH FIT, Water and Sanitation for Health Facility Improvement Tool.

### Stage 3: Initiation Workshop

We held an initiation workshop with approximately 40 key stakeholders, who were elected officials from municipal government (e.g., mayor, deputy mayor), bureaucrats and technical experts (e.g., WASH engineers, information technology specialists), representatives from NGOs working locally on WASH, leaders from participating health care facilities, and the media. During the workshop, we explained that the project aimed to develop an O&M policy for WASH in health care facilities and that costing would be done to develop budgets for the policy and provide a basis for evidence-based annual resource allocations.

The municipality formed a policy formulation committee, with the vice mayor as the formal chair, following their standard committee format. This committee was formed to steer the policy drafting process. A task force group comprised of 3 members—the municipality health coordinator, a local NGO representative participating in the project, and the municipality information technology officer—was formed to support the policy formulation committee. The task force supported tasks such as collecting and reviewing information and organizing consultations. Cost data collection and budgeting activities were conducted by the international NGO Terre des hommes and the local NGO Geruwa, with leadership from Thakurbaba Municipality. The Water Institute at the University of North Carolina at Chapel Hill provided technical support.

### Stage 4: Preliminary Costing

We met with each health care facility; participants included the health care facility in-charge, storekeeper, and nursing and support staff. During the meeting, the data collection team and meeting participants listed the type (e.g., pit latrine vs. pour-flush latrine) and quantity of all WASH infrastructure available at the health care facility. For each piece of infrastructure, health care facility staff were asked to describe its functionality in terms of the number of breakdowns per year and average duration of breakdown. We cross-referenced this list of infrastructure with our prepopulated costing spreadsheet and revised as necessary. An engineer estimated the costs of installation for all infrastructure (capital hardware and software) based on recall and records that were readily available for the costs of similar items being constructed. Health care facility staff estimated the specific products, quantities, and costs for maintenance, recurrent software, personnel, consumables, and support based on recall—again cross-referencing our prepopulated spreadsheet and revising as necessary. All estimates were discussed collaboratively with staff during the meeting to generate the best consensus estimate.

We used these line items to calculate current costs. Additionally, health care facility staff were asked to describe the additional infrastructure, goods, and services that they needed to achieve and sustain WASH services. These items, and their associated quantities and unit prices, were added to the spreadsheet to estimate the additional investment needed.

### Stage 5: Sharing and Discussion Workshop

We held a 2-day workshop to share and discuss the findings from preliminary costing. On the first day, we presented an overview of the costing spreadsheet and the preliminary data to the health care facility in-charges and the municipality health coordinator. Health care facilities agreed to conduct a second round of costing to improve the accuracy of preliminary costing and establish a system for routine cost monitoring. During the second day, the mayors and other municipality staff joined the meeting. We again presented an overview of the costing spreadsheet and preliminary data. We discussed possible elements of the O&M policy, given preliminary costs.

Municipal officials critiqued preliminary cost estimates as being too high and requested that health care facilities formally certify the data. We refined the costing spreadsheet to allow for additional space for health care facilities to provide comments as a form of budget justification. We finalized the costing spreadsheet with approval from the municipal government and the project committee, which endorsed a second round of “formal” data collection where health care facility leaders were asked to officially certify the accuracy of the data.

### Stage 6: Data Validation and Certification

We revisited all health care facilities and again explained the purpose of costing. This round, a broader range of staff was included in the meetings to triangulate the accuracy of data. Health care facility in-charges identified and delegated knowledgeable staff to review and revise costs as necessary, using the same bottom-up costing process. After data were collected, the project team conducted a second visit to present and review the data, at which time the health care facility staff verbally endorsed its accuracy. Health care facility in-charges then provided the final data and a signed letter certifying its accuracy.

### Stage 7: Budget Calculations

Using Excel spreadsheets, we estimated current costs and costs of additional upgrades necessary to reach basic service. A small number of essential line items for capital hardware were missing from the certified data for some facilities, notably for drainage, waste processing areas, autoclaves, and/or fencing. In these cases, we imputed using the mean cost from other facilities. The final spreadsheets with all line items for each facility are included in the Supplements and imputed data are notated for transparency.

We annualized capital hardware and capital software costs as the equivalent annual costs, using the time period as the estimated lifespan of the infrastructure in years (determined in consultation with the health care facility by an engineer from Geruwa, the local NGO supporting the project) and an annual interest rate of 8%.^15^^,^^16^ These expenses represented large 1-time investments to achieve WASH services (e.g., installation of infrastructure and start-up trainings for O&M), which are often financed through loans and repaid in installments. The annualized cost approximates the annual repayment amount.

Costs for capital maintenance, recurrent training, personnel, consumables, and support were routine expenses paid out of health care facilities’ normal annual operating budgets. These were already estimated as annual costs and required no further calculation.

We collected data in Nepalese rupees. Costs recorded in the Supplements are in Nepali rupees (NPR). Costs reported in this are converted to U.S. dollars (US$1=NPR130.5).

### Stage 8: Operations and Maintenance Policy Finalization and Approval

The policy formulation committee drafted an O&M policy for WASH in health care facilities based on the following information provided by the task force: (1) discussions with health care facility users as “rights holders” entitled to safe health care, civil society organizations, and duty bearers for ensuring adequate WASH (i.e., health care facility staff and management committees); (2) discussion and suggestions from the sharing and discussion workshop in Stage 5; and (3) results of budget calculations in Stage 7. This information was used to create a draft policy based on a standard template from the municipality.

The draft was then reviewed by project team members (i.e., representatives from the NGOs Terre des hommes and Geruwa) and the municipal WASH coordination committee, who provided feedback. The policy formulation committee incorporated this feedback into a final draft, which was submitted by the deputy mayor, on behalf of the committee, to the municipal assembly meeting. The municipal assembly, headed by the mayor, approved the policy.

### Stage 9: Dissemination and Advocacy for Scale-Up

At the municipal level, the municipality conducted dissemination workshop to ensure that all health care facilities were aware of the new policy, including newly established funds for operations and maintenance.

At the district level, we held a half-day dissemination and advocacy workshop with health care facility in-charges, representatives from 7 other municipal governments (mayors, deputy mayors, chief administrative offices, and health coordinators), local NGOs, the district health authority, and representatives from the media. We provided an overview of the costing and policy development process to raise awareness, encouraged replication in other municipalities throughout the district, and emphasized the importance of O&M. Other municipalities showed interest in conducting similar work, and Thakurbaba municipality officials spoke about their positive experiences and encouraged others to follow the process as well.

At the provincial level, Thakurbaba municipality organized a similar workshop on July 30, 2023, where the Chief Minister of the Lumbini Province was the chief guest. During this workshop, the municipality officials shared their best practices and learnings from O&M policy development and advocated for its replication in other municipalities in the province and allocations of funds for WASH in health care facilities O&M from the provincial government. Similar workshops are planned at the national-level for dissemination, but—at the time of writing—have not yet occurred. Nevertheless, as part of the advocacy efforts, the policy has been shared with relevant ministries, such as the Ministry of Water Supply and the Ministry of Health and Population, as well as with development agencies that are part of the National WASH Technical Working Group in Nepal. Terre des hommes Nepal has also shared the O&M policy development processes and learnings in an international webinar.

### Ethical Approval

This study was classified as not human subjects research by the Institutional Review Board of the University of North Carolina at Chapel Hill. Study activities received permission from local authorities of Thakurbaba municipality and from the in-charges of each health care facility.

## RESULTS

### Costs of Basic Service Provision

The Supplements include spreadsheets complete with all line items for each facility. Table 3 indicates the upfront capital hardware and software investments needed to achieve basic service; Table 4 indicates annual O&M costs and estimates of annualized capital costs. Table 5 disaggregates annual costs by WASH service.

**TABLE 3.** Upfront Capital Investments Required to Install WASH Services, Thakurbaba Municipality, Nepal

	**Health Post**				**Basic Health Center**			
	**Neulapur**	**Bagnaha**	**Shivapur**	**Thakurdwara**	**Ranipur**	**Bankatti**	**Mohanpur**	**Thakurdwara**
	US$							
Capital hardware	43893	38330	38347	15954	24629	10123	21379	21448
Capital software	368	368	460	1149	368	368	368	1149

Abbreviation: WASH, water, sanitation, hygiene, cleaning, and waste management.

**TABLE 4.** Annualized Costs for Capital Investments and Operations and Maintenance of WASH in Health Care Facilities in Thakurbaba Municipality, Nepal

	**Health Post**				**Basic Health Center**				**All**
	**Neulapur**	**Bagnaha**	**Shivapur**	**Thakurdwara**	**Ranipur**	**Bankatti**	**Mohanpur**	**Thakurdwara**	
Annual outpatient visits, no.	17,016	8,004	3,558	7,992	2,315	3,937	5,104	2,525	50,451
Current annual expenditure, US$									
Capital hardware^a^	4341	3607	3723	1564	503	733	984	1170	16625
Capital software^a^	0	0	0	117	0	0	0	117	234
Capital maintenance	1728	1498	2696	2018	845	1368	1516	1596	13265
Recurrent training	0	0	0	0	0	0	0	0	0
Consumables	1476	920	1616	1158	1048	670	808	922	8618
Personnel	0	0	0	0	747	0	0	0	747
Support	0	0	0	0	0	0	0	0	0
Additional annual investment needed to reach basic service, US$									
Capital hardware^a^	102	102	84	113	2470	274	1089	972	5206
Capital software^a^	37	37	47	0	37	37	37	0	232
Capital maintenance	184	184	156	153	153	184	153	153	1320
Recurrent training	755	648	252	578	578	578	578	578	4545
Consumables	315	193	547	529	457	447	441	838	3767
Personnel	274	274	403	274	274	274	274	473	2520
Support	316	316	170	363	316	316	316	162	2275
Summary costs, US$									
Total annual capital costs^b^	4480	3746	3854	1794	3010	1044	2110	2259	22297
Total annual O&M costs^c^	5047	4033	5841	4593	4417	3837	4086	4722	36576
Total annual cost for basic service	9527	7779	9695	6387	7427	4881	6196	6981	58873
Average cost per outpatient visit	0.56	0.97	2.72	0.80	3.21	1.24	1.21	2.76	1.68

Abbreviations: O&M, operations and maintenance; WASH, water, sanitation, hygiene, cleaning, and waste management.

a Capital hardware and capital software costs are annualized from the total upfront investment cost, using an interest rate of 0.08.

b Total annual capital costs include capital hardware and capital software.

c Total annual operations and maintenance costs include capital maintenance, recurrent training, consumables, personnel, and support.

**TABLE 5.** Total Annual Costs for Disaggregated by WASH Service, Thakurbaba Municipality, Nepal

	**Health Post**				**Basic Health Center**			
	**Neulapur**	**Bagnaha**	**Shivapur**	**Thakurdwara**	**Ranipur**	**Bankatti**	**Mohanpur**	**Thakurdwara**
	US$							
Water	1017	619	1068	781	654	527	577	680
Sanitation	2589	1413	1660	1197	1040	752	1123	932
Hygiene	226	146	375	45	253	220	296	67
Waste management	483	348	387	277	1069	160	121	100
Cleaning	2681	1788	3541	2324	1457	1582	1667	2225
Other^a^	2532	3466	2664	1763	2955	1640	2412	2537

Abbreviation: WASH, water, sanitation, hygiene, cleaning, and waste management.

a Includes fencing and costs that were shared across multiple categories (e.g., infection prevention training, operations and maintenance training common to all infrastructure).

The current annualized cost for all basic WASH services ranged from US$2771 to US$8035 per facility per year. The largest single contributor to annual costs was capital hardware. However, the combined annual O&M costs (capital maintenance, recurrent training, consumables, personnel, and support) exceeded annualized capital costs in all facilities.

Additional investment needed to achieve basic service for all cost categories ranged from US$1659 to US$4285 per facility per year. The areas of greatest need were consumables, recurrent training, and capital software. All facilities reported needing additional waste management and cleaning supplies to reach targets for basic service. All facilities identified additional line items for recurrent trainings were needed to reach basic service. Few facilities had current expenditure on personnel and included line items for support staff and O&M focal persons as additional expenditures needed to reach basic service.

Additional investment needed to achieve basic service for all cost categories ranged from US$1659–US$4285 per facility per year.

On average, the highest annual expenditure was for cleaning, then sanitation. Cleaning costs were driven primarily by consumables (e.g., detergents, mops, brooms) and personnel salaries to perform cleaning activities. Facilities with maternity services had particularly high cleaning costs. Sanitation costs were driven more by capital hardware and capital maintenance costs.

### Policy Outcomes

On March 25, 2023, Thakurbaba municipality formally adopted an O&M policy for WASH in health care facilities. The policy calls for the establishment of a recovery fund that can be used for WASH infrastructure repair and maintenance at any municipal health care facility. The amount allocated to the fund is not formally specified in the policy but has been agreed through meetings of the municipality general assembly as US$3831 (NPR500,000), which has been allocated from the municipality’s discretionary funds. The fund does not have an expiration date, and the municipality aims to replenish the fund at the end of every fiscal year (or sooner if all funds are exhausted). The policy also aims to establish an additional recovery fund of US$383 (NPR50000) in each health care facility for minor repairs and maintenance of WASH infrastructure. At the time of writing, US$153 (NPR20,000) per health care facility had been allocated.

In Nepal, individual municipalities have the authority to allocate resources as needed, including for O&M. As such, the following provisions were made by Thakurbaba municipality for O&M of WASH services in their health care facilities. Individual health care facilities may spend directly out of their dedicated facility-level recovery fund of US$153 (NPR20000). The spent amount is replenished from the municipality as needed. Individual health care facility recovery funds are jointly overseen by the chairperson of the health care facility operation and maintenance committee and the health care facility in-charge.

For larger amounts, health care facilities must send a request to spend from the municipal-level recovery fund of US$3831 (NPR500000), which is reserved for WASH in health care facilities O&M. Requests are sent using existing procurement forms and processes. Smaller expenditures can be approved by committees within the health care facility (e.g., WASH FIT committees, O&M committees). Larger expenditures must be recommended and approved by the municipal WASH coordination committee. The chief executive officer of the municipality (or individual assigned by them) is responsible for maintaining the records of decisions and expenditures from the municipal-level recovery fund.

For minor O&M tasks, repairs may be handled in-house by cleaning and maintenance staff. The new O&M policy has opened a way to train and mobilize these staff. For major O&M tasks, the recovery funds can be deployed to hire private sector contractors. Qualified technicians are available locally, such as engineers in the municipality. As part of broader efforts to strengthen WASH in health care facilities in Thakurbaba municipality, WASH FIT committees had been previously established in each health care facility. Budgets created as part of this project include line items for annual WASH FIT training, which is designed to ensure that WASH FIT teams remain in place with authority and capacity to identify and execute O&M activities.

The municipality also adopted a guideline called the “WASH in health care facilities O&M fund implementation procedure,” which outlines expected costs of WASH infrastructure and O&M (including necessary tools, parts, supplies, and personnel) and describes procedures for implementing O&M activities. At present, there are no specific targets for infrastructure coverage or functionality, though targets may be established in future based on ongoing assessments and development of national guidelines.

### National Advocacy Outcomes

At the national level, we shared an interim report on costing from this project with the team at the Ministry of Health and Population that is involved with preparing the “National Roadmap for Water Sanitation and Hygiene of Health Care Facilities in Nepal.” The costing report has been included in the references as evidence to inform the national roadmap. A draft national roadmap has been shared with stakeholders for consultation, and—at the time of writing—was nearing completion.

## DISCUSSION

We conducted costing and advocacy activities in Thakurbaba municipality, Nepal, with the aim of developing budgets and an O&M policy for WASH in health care facilities. Through a joint exercise with health section and municipal government actors, we successfully collected cost data in 8 health care facilities, which were used to draft budgets for annual costs of WASH service provision. These budgets informed an O&M policy, which was formally adopted by Thakurbaba municipality. The municipal government has established a recovery fund of US$3,831 across the facility—and an additional US$153 per facility—to implement the policy. The municipal government also has a plan to advocate for the adoption of a similar policy at the national level. The calculated costs of WASH provision have also been used to inform the nationally costed roadmaps for WASH in health care facilities.

The budgets drafted from the collected cost data informed an O&M policy that the Thakurbaba municipality formally adopted.

While there have been previous studies costing WASH in health care facilities,^14^^,^^17^^–^^19^ to our knowledge this is the first study to document the application of cost data for improved policy and practice. Even where cost evidence is being generated, its translation into meaningful improvements in policy and practice has been slow. Collecting cost data in isolation is of limited value if those data cannot be translated into improved policy or practice. As of 2024, only 16% of countries reporting data for progress on the Eight Practical Steps have developed national coordination mechanisms and costed roadmaps.^2^

Prior studies have suggested 4 factors that can improve the translation of evidence into policy and practice: salience, credibility, legitimacy, and timeliness.^20^^–^^22^ Salience refers to evidence that is highly relevant to critical health issues within the context. Credibility refers to the rigor or scientific credibility of the evidence. Legitimacy refers to the process of producing the information and whether it has been balanced, fair, and respectful of stakeholders’ values. Timeliness refers to the time between generating evidence and disseminating it to the relevant policymakers. We reflect on lessons learned during our project that demonstrate these factors. We discuss the barriers and facilities to budgeting and advocacy that we experienced in Nepal and reflect on how our process may be adapted for other sociopolitical contexts.

### Salience: Adapt Costing Tools to Reflect Local Needs and Standards

During our pilot testing, we assessed the resources that were used locally by health care facilities to provide basic WASH services. While we referenced the JMP service levels, we also incorporated line items for infrastructure, goods, and services that health care facilities identified as locally relevant needs. This helped ensure that final budgets reflected salient WASH needs in the local context. Local civil society organizations and NGOs can often be helpful in ensuring that a project reflects local needs, standards, and cultural preferences. Those local stakeholders will also be in a position to sustain the efforts once the international stakeholders have finished with a technical project.

Needs for WASH services will vary by facility type—both within Nepal and other countries—and over time, accounting for population changes, changing weather and climate conditions, and other external variables. We conducted this project in smaller, rural facilities providing predominantly primary care, and our benchmark was basic WASH services. Facilities providing more advanced service levels (e.g., tertiary hospitals) will require different—and likely more expensive—budget line items to achieve a suitable WASH service level.^12^^,^^13^ Yet these more advanced service levels and associated resource inputs are not well documented in prior studies.^19^ As such, teams attempting to replicate this budgeting process in other health care facility types will likely need a more intensive piloting phase to better understand required line items. Existing costing toolkits contain guides for formative research and piloting to document line items,^13^ which could support this step. Tools such as the WHO and UNICEF’s Water and Sanitation for Health Facility Improvement Tool (WASH FIT)^23^ can help identify improvement needs and associated line items, but WASH FIT is also designed for smaller, primary health care facilities and may also require adaptation.

Standards for WASH services—and by extension costs to meet those standards—will also vary by context. In Nepal, governance is highly decentralized, and municipal governments have a high degree of autonomy to set targets and regulate WASH services and the health system overall.^8^^,^^9^ As such, we did not reference national or regional guidelines. However, these may also be relevant information sources in other settings with stronger centralization. In countries where standards for WASH in health care facilities are nationally established and regulated, we recommend tailoring budgets to those standards. At the policy advocacy stage, the status of decentralization in a given country must also inform the direction of the advocacy effort: at what level do officials have the authority to enact stronger policies and increased budgets for WASH in health care facilities?

### Credibility: Include a Variety of Stakeholders in Costing

During costing, we incorporated a variety of staff in meetings to estimate costs to enhance credibility. Health care facility staff were knowledgeable about costs of O&M (capital maintenance, recurrent training, personnel, and support) but struggled to estimate capital costs. We invited an engineer to participate in costing, who was more knowledgeable on construction costs for capital hardware. This reflects experiences with costing in other settings, which have found that knowledge of costs is highly compartmentalized and that including staff from different roles improves accuracy.^12^^–^^14^ Triangulating results between different perspectives is also a well-accepted practice to improve data quality in many research disciplines.^24^^,^^25^

Including staff from different roles improves costing accuracy as knowledge of costs can be highly compartmentalized.

We asked health care facility in-charges and senior leadership staff to help identify knowledgeable individuals to participate in and triangulate data collection. In other contexts, procurement of goods and services related to WASH in health care facilities may not be coordinated within individual health care facilities but rather centralized within larger administrative units (e.g., secondary or tertiary health care facilities that receive and distribute supplies to smaller facilities). Some countries also nationally or regionally control prices for medical goods—for example, through standard price lists and distribution from a network of nationally-managed warehouses.^26^ In these instances, costing tools require less tailoring to the local context and should instead reflect the standard prices and procurement systems. Project teams should understand local systems for logistics and procurement before engaging in budgeting; assessments can be guided by existing resources.^12^^,^^13^

### Credibility and Legitimacy: Build Trust and Transparency for Data

We presented the results of preliminary data collection in a sharing and discussion workshop. During this workshop, municipal government officials critiqued the accuracy (i.e., credibility) of data. In response to this, we initiated a second round of data collection to address these concerns (i.e., Stage 6: data validation and certification). To address credibility concerns, the municipal government asked health care facilities to officially certify the data in a signed letter. By creating a formal certification process, this created pressure on health care facilities and compelled a wider range of knowledgeable staff to participate in data collection and verify its accuracy.

Certification also improved legitimacy. The sharing and discussion workshop allowed stakeholders to voice their concerns about the data collection process and results, and we modified our approach and incorporated a step for health care facilities to formally certify the data before it was used for budgeting and policy development. This certification process addressed stakeholder concerns and helped improve the legitimacy of the data.

In other contexts, different mechanisms may be more appropriate to improve credibility and legitimacy. For example, systematic reviews on effective health advocacy recommend making data public to enhance transparency.^27^ In Nepal, we invited journalists and local media to participate in workshops. However, in other contexts, particularly those with less trust in journalism and media, may benefit from disseminating through other means. Patient advocacy groups, for example, have a long, influential history in health advocacy campaigns^28^ and may be a trusted partner to vet and endorse data. Seeking and incorporating feedback is another recommended practice for shaping effective advocacy messages.^29^ In Nepal, we did this primarily through workshops and committees, but in other contexts individual engagement of key decision-makers may also be necessary. Associations of health workers (e.g., nurses, doctors, other health workers) may also be helpful in enhancing credibility and legitimacy. More broadly, establishing ground rules for the outputs (e.g., indicators measured and populations sampled) and processes for data collection (e.g., ethics, consent, and confidentiality) should be agreed upon by evidence generators and policymakers developing the evidence-based policy.^27^^,^^30^ Agreements may begin as informal discussions but are strengthened by formal agreements, such as memoranda of understanding or terms of reference.

### Legitimacy

#### Engage Policymakers in Evidence Generation

Early in the project, the municipality created a policy formulation committee chaired by the vice mayor and a task force to support the committee. This policy formulation committee did not participate in day-to-day activities for data collection but was engaged in sharing and dissemination workshops to receive updates after major data collection milestones. Forming the committee was important to show the municipal government’s support and approval for the project and endorse data collection activities. It also ensured that the advocacy campaign was aware of—and built on—the existing policy framework (or lack thereof), and that the campaign was well aligned with the government’s own policy and budget timelines. Keeping municipal government stakeholders informed via the task force was also important for transparency; building trust in the data; and ensuring that the municipality’s needs, concerns, and priorities were being addressed. All of these steps make successful policy and budget outcomes more likely.

Keeping municipal government stakeholders informed via the task force was also important for transparency; building trust in the data; and ensuring that the municipality’s needs, concerns, and priorities were being addressed.

Before presenting evidence to policymakers, evidence should be tailored. Considerable guidance has been written on tailoring evidence to policymakers for advocacy, and an in-depth review is beyond the scope of this article. Briefly, good practices include: avoid jargon, assume the audience is nonspecialist but not ignorant, communicate clearly and succinctly, and ensure that systems are in place for questions and follow-up.^27^^,^^29^^–^^31^ Evidence on effective health advocacy also suggests that narrative and storytelling can be compelling forms of evidence for policymakers.^32^^,^^33^ WASH in health care facilities is key for maternal and child health,^34^ and narratives and storytelling centered around the experiences of women, children, and other vulnerable patient populations may prove particularly influential.^33^ It is also advisable to recognize that the targets of an advocacy campaign are often political leaders, either elected or appointed. They are concerned about providing desired services—including WASH in health care facilities—to their constituents, in a timeframe subject to electoral cycles.

#### Target Advocacy Efforts at the Right Level

In Nepal, the shift to federalism has empowered municipal governments to oversee basic and essential care. As a result, primary health now falls within the jurisdiction of local authorities (i.e., municipalities), and they have strong autonomy over local policymaking and budgeting for WASH in rural health care facilities.^7^ Thus, these were the key stakeholders we engaged on policy development and advocacy efforts. However, in countries with more centralized governance structures, advocacy may more effectively target decision-makers at the national or regional level. For example, WASH and environmental health in health care facilities may be regulated by multiple ministries for health, environment, and rural development—with key decision-makers across multiple ministries and complex relationships between each. Advocacy guides recommend and provide detailed instruction on identifying stakeholders and targets for advocacy messages.^35^^,^^36^ Regardless of what decision-makers are identified, advocates should anticipate turnover of elected officials or bureaucrats who are reassigned and be prepared for ongoing efforts to engage decision-makers.^27^ Advocacy efforts should, therefore, expect and plan for a multiyear process and should target both elected and/or appointed policymakers with finite political mandates and technical leaders who are likely to remain in place for longer periods of time.

We anticipate that different government structures will present different opportunities and challenges. In the decentralized Nepali governance system, we found that local government officials had limited technical knowledge of WASH and required more orientation to the background, importance, and standards of WASH in health care facilities. With fewer layers of government, there were fewer stakeholders to engage, and government officials had more autonomy to act local changes quickly. However, scaling up across Nepal’s 753 municipality and rural-municipality governments will require substantial effort. In contrast, we hypothesize that more centralized systems of government will be more complex, in part because of the need for wider consultation when making new policies or changing existing policies. This more complex process is likely to be slower but require less effort for scaling up. For example, case studies examining advocacy and policy change at the national level often measure progress against a 5, 10, or 20-year timescales,^37^ whereas we initiated our project in Thakurbaba municipality in 2022, and the O&M policy was adopted in early 2023.

### Timeliness: Establish Systems for Ongoing Data Collection

We collected data by asking health care facility staff to estimate costs. These estimates were based in part on recall of prior expenses. During interim sharing and discussion workshops, stakeholders suggested adapting the data collection tools to an online dashboard. The project team selected KoboToolbox connected to online Power BI (a data visualization software) as the preferred data collection platform because of its simplicity, the team’s prior knowledge, and its fitness for purpose. The online dashboard is being established and piloted in 2 health care facilities. It will collect periodic data on the functionality of all the WASH facilities, use of selected infrastructure (e.g., handwashing stations), preventive maintenance activities, repairs (e.g., to damaged infrastructure), and the cost and response time of repairs. This database is intended to help develop appropriate strategies and targets for improving functionality and use and to reduce the cost on O&M. Delivering this information through an online dashboard will improve the availability and timeliness of information for decision-makers.

Advocacy campaigns designed to translate costs data into policies and budgets often take years. To ensure timeliness, costing data should continue to be collected and revised over a multiyear timeframe, to account for changing costs of inputs (both capital investments and O&M costs), population growth and movement (e.g., increased patient load), and changing climate and weather conditions.

### Limitations

We estimated costs using bottom-up costing. Health care facility staff estimated the quantity and unit prices of resources used in WASH provision based on available records and their best recall. While we triangulated estimated costs between different staff members and asked health care facilities to certify data to improve accuracy, we still found that certified data were missing a small number of key line items. We imputed missing data for capital hardware that were essential to reach the JMP basic service level (e.g., autoclaves). However, there are currently no comprehensive guidelines on other cost categories (e.g., quantity of consumable products like soap needed for adequate cleaning).

Bottom-up costing is naturally prone to underestimates, as it relies on a comprehensive accounting of line items to generate accurate estimates. As such, the true costs of WASH service provision presented here may be underestimates. However, we referenced prior studies of essential resources for WASH provision and imputed the most expensive missing line items for capital hardware, so we propose that any missing line items would have only a marginal effect on cost estimates. Costs that occur of premises for the health care facility—notably, aspects of the sanitation service chain for sewage treatment after desludging—were not included in this study. We also did not consider potential revenue generation associated with WASH services. For example, health care facilities in Nepal receive a stipend for every delivery—which would offset the costs of cleaning—that we did not consider.

## CONCLUSIONS

A supportive policy environment and adequate funding are essential to achieving and maintaining WASH in health care facilities, and developing nationally costed roadmaps is recommended by the WHO and UNICEF as part of the Eight Practical Steps to achieving universal access to WASH in health care facilities.^1^ Nepal has made considerable progress on steps for assessing conditions, documenting needs, and setting targets. However, developing budgets and allocating funding for long-term O&M remains a challenge. We conducted costing and advocacy in Thakurbaba municipality, Nepal, to accompany the development of an O&M policy for WASH in health care facilities.

Our efforts resulted in successful development and adoption of an O&M policy for WASH in health care facilities by Thakurbaba municipality. At the time of publication, advocacy efforts to replicate this process were underway in other municipalities, and findings on costs had been incorporated into a draft nationally costed roadmap. Cognizant that every effort to translate evidence into policies and budgets is unique and context-specific, we propose that the process described in this article can be used as an example to guide progress toward universal access to quality WASH services in health care facilities in other settings—particularly following the framework of the Eight Practical Steps.

## Supplementary Material

GHSP-D-23-00491_supplements.zip

## Notes

**Peer Reviewed**
